# News and Perspectives: Words matter in primatology

**DOI:** 10.1007/s10329-023-01104-6

**Published:** 2023-11-30

**Authors:** Michelle Bezanson, Liliana Cortés-Ortiz, Júlio César Bicca-Marques, Ramesh Boonratana, Susana Carvalho, Marina Cords, Stella de la Torre, Catherine Hobaiter, Tatyana Humle, Patrícia Izar, Jessica W. Lynch, Tetsuro Matsuzawa, Joanna M. Setchell, Gladys Kalema Zikusoka, Karen B. Strier

**Affiliations:** 1 Department of Anthropology, Santa Clara University, Santa Clara, CA USA; 2 Department of Ecology and Evolutionary Biology, University of Michigan, Ann Arbor, MI USA; 3 Escola de Ciências da Saúde e da Vida, Pontifícia Universidade Católica do Rio Grande do Sul, PUCRS, Porto Alegre, RS, Brazil; 4 Mahidol University International College, Nakhon Pathom 73210, Thailand; 5 Primate Models for Behavioural Evolution Lab, Institute of Human Sciences, University of Oxford, Oxford, UK; 6 Gorongosa National Park, Sofala, Mozambique; 7 Department of Ecology, Evolution and Environmental Biology, Columbia University, New York, NY 10027 USA; 8 Universidad San Francisco de Quito, Quito, Ecuador; 9 Wild Minds Lab, School of Psychology and Neuroscience, University of St Andrews, St Andrews, UK; 10 Durrelll Institute of Conservation and Ecology (DICE), School of Anthropology and Conservation, University of Kent, Kent, UK; 11 Department of Experimental Psychology, University of São Paulo, São Paulo, Brazil; 12 Department of Anthropology, and Institute for Society and Genetics, University of California, Los Angeles, CA 90095 USA; 13 Division of the Humanities and Social Sciences, California Institute of Technology, Pasadena, CA 91125 USA; 14 Department of Pedagogy, Chubu Gakuin University, Gifu, 504-0837 Japan; 15 Shaanxi Key Laboratory for Animal Conservation, College of Life Sciences, Northwest University, Xi’an, 710069 China; 16 Department of Anthropology, Durham University, Durham, UK; 17 Conservation Through Public Health (CTPH), Entebbe, Uganda; 18 Department of Anthropology, University of Wisconsin-Madison, Madison, WI USA

**Keywords:** Colonialism, Language, History

## Abstract

Postings on social media on Twitter (now X), BioAnthropology News (Facebook), and other venues, as well as recent publications in prominent journals, show that primatologists, ecologists, and other researchers are questioning the terms “Old World” and “New World” due to their colonial implications and history. The terms are offensive if they result in erasing Indigenous voices and history, ignoring the fact that Indigenous peoples were in the Americas long before European colonization. Language use is not without context, but alternative terminology is not always obvious and available. In this perspective, we share opinions expressed by an international group of primatologists who considered questions about the use of these terms, whether primatologists should adjust language use, and how to move forward. The diversity of opinions provides insight into how conventional terms used in primatological research and conservation may impact our effectiveness in these domains.

## Introduction

Primatologists have joined other scholars and the public in discussion about the terms “Old World” and “New World” because these terms reflect both a colonial history and continued neocolonial practice. There is substantial debate about whether the terms should be replaced by less politically loaded and offensive terms. The ways that humans employ and adapt language can rapidly shift the function and meaning of words on each occasion of their use and in their broad generalized meanings. Consider new terms from the COVID-19 pandemic. In 2020, society learned about the English terms bandwidth, flattening the curve, herd immunity, contact tracing, and pods. Some of these terms have already disappeared from general use, while others may remain for centuries. As primatologists, we can therefore ask the question: Have the terms “New World” and “Old World” lost their colonial meaning in current usage? If so, how? If not, is the continued use of these terms offensive, and if so, what does this mean for primatology or other disciplines that use the terms?

## History

**Fig. 1:**
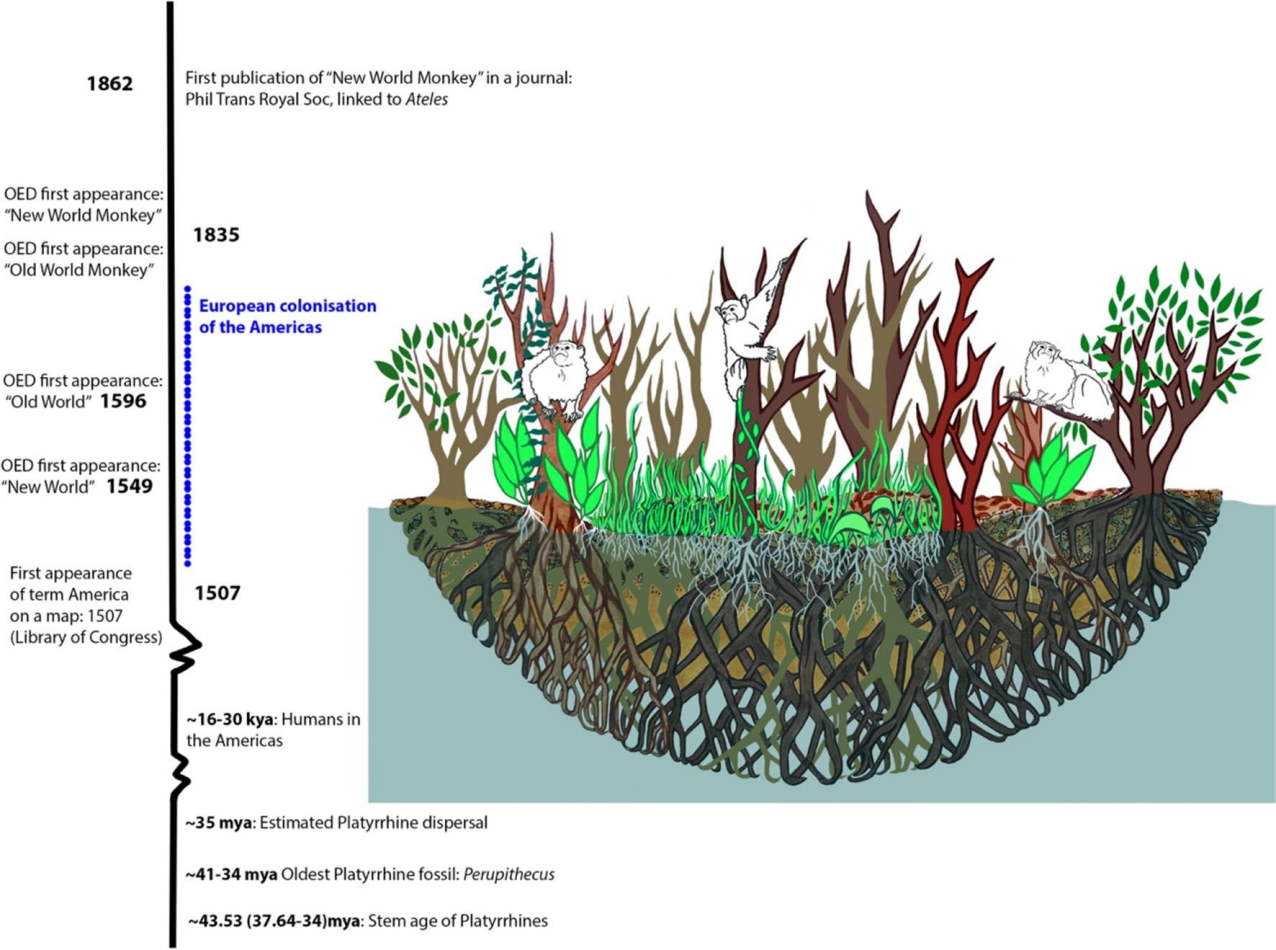
Timeline of Platyrrhine origins and term origins. The illustration shows one interpretation of the dispersal method of stem monkey(s) from Africa rafting toward South America. Citations: Bond et al. (2015), Potter et al. (2018), Becerra-Valdivia and Higham (2020), Seiffert et al. (2020), New World/Old World Oxford English Dictionary (OED) online (2023)

In 1885, William Swainson described the monkeys of the Americas and Afroeurasia, respectively, as monkeys of the “New World” and the “Old World.” These terms “New World” and “Old World” first arose in the 1500/1600s and were used by European explorers as they described how they conquered new lands, conquered Native peoples, and sought new riches (Oxford English Dictionary, May 10, 2023) (Fig. 1). The terms were used by early Spanish, Italian, and British explorers in descriptions of “discovery,” “plundering,” and “exploiting” the lands and Indigenous peoples of the Americas. In academic texts and conversations, the terms are commonly used to describe features of monkeys found in the Americas or Afroeurasia. For example, monkeys of the Americas (Platyrrhini) are characterized by three premolars, differing cranial/nasal features, and some have prehensile tails, whereas the monkeys of Afroeurasia (Catarrhini) are characterized by two premolars, relatively narrow nasal openings, and some have ischial callosities. Academically, the terms Old and New may also reflect the human/non-human primate dispersal to the Americas, but they arose with colonial goals in a colonial context. While it is believed from a Western scientific perspective that ancestral Platyrrhini arrived in the Americas via sweepstakes dispersal from Africa, it should also be noted that some of the earliest fossils of primates or primate-like mammals (i.e., *Purgatorius*, some *Teilhardina*, and *Notharctus*) are from North America, somewhat complicating this “Old” to “New World” story (Fleagle 2013; Lynch Alfaro 2017). Additionally, it is from Western scientific evidence that anthropologists agree that humans dispersed from Africa throughout the world and only “recently” to the Americas, but in many American cultural traditions there is a different origin story and history to humans and their relationship to the land in the Americas.

Primatologists, ornithologists, ecologists, biologists, and other scholars have been discussing the appropriateness of using “New World” (NW) and “Old World” (OW) to refer to species originating in the Americas versus Africa, Asia, and Europe (e.g., Adame 2023). Contributors to this commentary have either engaged in these conversations on social media and/or in professional settings. Some journals and textbooks have either changed the language to other options (e.g., the *International Journal of Primatology* now asks authors to avoid the use of these terms and instead use formal taxonomic terms, American and Afroeurasian monkeys, or other similar terms) or have explained the controversy, history of terminology, and alternatives to the terminology (e.g., Strier 2021). Because primatology is an international field of scholars and we do not want to be colonial in our attempts to be less colonial (e.g., Hirschfeld et al. 2023; Lewis 2023), we (Bezanson, Cortés-Ortiz, and Strier) posed questions of OW/NW term use to an international group of scholars including elected officers of the International Primatological Society (IPS) in 2018–2020 and other colleagues who attended and were active in the roundtable scheduled for the 2020 joint meeting of the IPS and the Sociedad Latinomericana de Primatología (SLAPrim). Due to the impact of COVID-19, the roundtable was separated into a session held virtually in August 2021 and a hybrid in-person/virtual component during the meeting in Quito, Ecuador in January 2022. The resulting commentary is a summary of ideas presented by the participants at one or both sessions.

## The conversation

“Colonialism” is domination of physical space and “Decolonization” has the ultimate goal of repatriating Indigenous lands and life (Bezanson et al. 2023; Rodrigues et al. 2022; Tuck and Yang 2012; Waters et al. 2022). By discussing potential changes in language, naming, and scientific nomenclature in primatology (as in any other field), we seek to make our field more inclusive. Words can be considered subtle forms of discrimination (microaggressions), which can cause exclusion and drive emerging professionals away from the discipline (Harrison and Tanner 2018). As primatologists, we extract resources in the form of data, recognizing that in many cases, primatologists are guests in environments where we have not originated (Riley and Bezanson 2018).

Academic goals to decenter colonial history are equally genuine and ambitious, but it is also important to consider that our science is primarily published in English, our focus is on terms that arose in the English language, and these same terms are also used in other colonial languages widely used in primatology, i.e., Spanish, Portuguese, French. English common-use terms and scientific terms may have different meanings and histories in other languages, and terms that presuppose values or are harmful to some individuals and may not be problematic to others (Adame 2023).

Given this context, we composed the following prompts for our virtual and hybrid panels.

## Prompts

Prompt for the August 2021 Virtual IPS-SLAPrim Virtual Program.

1. Do you use the language “New World” and “Old World” in your research?
2. Does the language “New World” and “Old World” suggest colonial history in your area or is this context something you have never previously thought about in your research and writing on nonhuman primates?
3. Given that this language is offensive to some individuals, do you or would you support an attempt to update these descriptors?
4. Can our choices of language influence conservation priorities in positive or negative ways?
5. Do you have any other ideas about decolonization in the history of primatology and use of terms about primates?

Following the August 2021 virtual event, we asked the participants of the January 2022 hybrid event to reflect on the conversations we had during the virtual event.

## Common Goals

As scientific professionals, we must recognize that our own conscious and unconscious biases, and language related to them, may have large impacts on our students and colleagues. Harrison and Tanner (2018:1)

All contributors agreed that our primatological goals involve inclusion, equity, making our field accessible, and a rigorous ethical approach in our research and information sharing. We agreed that primatology’s biggest challenge is preventing extinction (and population decline: see Dirzo et al. 2014; Estrada et al. 2017) of nonhuman primates. While we did not all agree on how we might move forward regarding language, the conversation was respectful, and we welcomed opportunities to continue the conversation and to learn from each other in the future. The following description reflects varying viewpoints from international experts who work in the Americas, Asia, and continental Africa (some of them citizens of those regions), on topics ranging from behavioral ecology, to cognition, conservation, evolution, and genetics.

**What do the terms “New World” and “Old World” mean to us and should primatologists work to replace the terms (prompts 1–3)?** All participants reported having used “New World” and “Old World” in their research and teaching. It was also suggested that primatology is not alone. Biology, ecology, archaeology, botany, viticulture, and anthropology textbooks all use these terms, and many of these fields are having similar discussions about the appropriateness of their use. One challenge raised is that English is the current language of science and the participants recognized that this is also a barrier to inclusivity. However, while all participants agreed that primatology should be more accessible and many participants on the panel routinely conduct their research in other languages and work to translate their science, we did not predict that the field would move away from English dominance soon. We could likely take on this topic in an entirely new panel and this fact filtered into our conversation many times.

Seven of 16 panelists have changed their teaching and scholarship to use different terms, and provide explanations of their reasons for doing so (Table 1). For example, one panelist has stopped using the terms but takes the opportunity to discuss these types of challenges in lectures. Because “New World” and “Old World” are ubiquitous in the texts, films, and other types of media that primatologists use while teaching, it is necessary to explain the reasons for using different terms. Another panelist noted that the same terminology can mean different things for different people, and this can depend on both their primary languages and culture. For this panelist, the terms “New World” and “Old World” are easy to say and remember and therefore convenient for teaching, and changing the terms could cause confusion. Additionally, a change in terminology takes time, and if some colleagues did not conform to the changes because of lack of awareness, it could negatively influence the perceptions of our colleagues. However, if “New World/Old World” terms are offensive to anyone, this would be enough of a reason to make the change. Language reflects our positionality, and as humans, we must recognize that language evolves, changes, and can hold multiple meanings across contexts of use. Three panelists acknowledged that they had been unaware of the controversy over “New World/Old World” terminology. In fact, among our panelists, many non-North American/non-English (as a first language) users did not initially see an issue with the terminology, but after their participation, they recognized it as a worthy issue to consider. While recognizing that terminology is an issue, several panelists maintained the view that there were other more pressing matters, such as primate conservation, than these two terms.

**Table 1:** Examples of ways in which primatologists refer to the monkeys that live in different regions of the world

Flat-nosed monkeys of North, Central, and South America	Downward-facing nostrils monkeys of Europe, Africa, and Asia	Arguments against/issues
New World monkeys	Old World monkeys	Colonial perspective/implications
Platyrrhines	Catarrhines (Alternative: Catarrhine monkeys) Cercopithecoidea/ Cercopithecids	Issue: Catarrhines includes the apes Hard to say and remember
Monkeys of the Americas American monkeys	Monkeys of Africa/Asia Afroeurasian monkeys	Issue: Too long The term America is taken from the name of a European colonizer
Monkeys of the Western Hemisphere	Monkeys of the Eastern Hemisphere	Issue: Too long. Confusing. The western part of Africa is included in “Western” and does not capture the correct geography
Neotropical monkeys	Paleotropical monkeys	Same connotation of “New” and “Old”; colonial perspective/implications

## Given that many English terms in science and conservation have nefarious backgrounds, how far do we go?

The conservation movement has been as damaging to Indigenous peoples as extractive industries. National parks, ecological restoration projects, and even the use of certain terms–especially ‘wilderness’, are associated with forced displacement of entire communities, erasure of Indigenous histories in education and public memory, economic marginalization, and violations of cultural and political rights. –Kyle Powys Whyte (2018)

The terms “New World” and “Old World” are not the only questionable terms in primatology. For example, the term “America” arose in the early 1500s from Florentinian Amerigo Vespucci, a European colonizer, despite the presence of humans in the Americas before his time. Many terms have questionable backgrounds and some might be shocked that many terms in widespread use have racist origins or are considered derogatory by Indigenous groups of North and South America. For example, the use of the term “powwow” (let’s have a gathering) or the expression “low on a ‘totem pole’” (status) when used to convey meanings unrelated to their origins is considered offensive by many individuals (Stollznow 2020). Nonetheless, one panelist suggested that just because some individuals find terms offensive, we cannot update every one of them unless, of course, plausible arguments support the need for change. There have been efforts to change English common-use names of primates and other organisms (Chen-Kraus et al. 2021; Driver and Bond 2021; Fairbanks et al. 2023; Kano and Nishida 1999; Rubis 2020). English common-use names are one avenue of (taxonomic) language that can be changed, with many researchers suggesting that we refer to Indigenous names whenever possible. Challenges with this approach include how and by whom it is decided which Indigenous names to use. For example, the term Orangutan—while often considered Malay in origin—has been suggested to have originated in language use resulting from Dutch colonial administration and is known by different local names in different parts of its range, such as Mawas, Mias, Maias, Mawih, Kihau, and Kogiu (Fairbanks et al. 2023; Rubis 2020).

Reverting to Indigenous names for other primates might be even more complicated where Indigenous populations have been replaced by immigrants from other places with different languages. As a result, Indigenous names may change in the same location over time. In these cases, while the use of Indigenous names recognizes historical precedence at the available recorded level, these names may be unfamiliar and even disassociated from the terms used by the people who now live in proximity to the primates. This is what happened when Brazilian scientists encouraged the use of the Indigenous name of muriquis instead of the Portuguese term, mono carvoeiro, which was not a translation of muriqui, but rather a description of its charcoal facial coloration. Initially, the local Portuguese-speaking farmers in the communities living near muriquis were not concerned with this history, but now they also refer to the monkeys as muriquis. Nonetheless, some writers who are unfamiliar with the importance of the origins of this name change continue to refer to muriquis in contemporary publications by the long discarded English common-use name of woolly spider monkey, which was not a translation of either muriqui or mono carvoeiro, but rather a reflection in English of the muriquis’ similarities to woolly monkeys and spider monkeys, neither of which is an Indigenous name.

Several participants suggested that we may be giving too much weight to these names. Although the terms “New World” and “Old World” arose reflecting the perspective of Europeans in the age of exploration and colonization, do these terms generally retain a connotation of colonialism today? It was proposed that instead they might act as names (they are proper nouns, written with uppercase first letters), and names that have now been used for centuries. At the August 2021 Roundtable, some colleagues from parts of the world that were once colonized were familiar with the terms as references to geographical regions or migration histories but had not previously associated them as referencing colonial history. Some associated them with the relative age of human civilizations, not with European exploration and colonization. So, the question remains: who, today, is bothered?

When the idea for this roundtable emerged, the majority of discussion on this topic was occurring in social media (Facebook, Twitter), where the individuals who were bothered were North American Indigenous researchers, students from North America, early career primatologists, and mid–later-career primatologists who are working on decolonizing our fields (see Bezanson et al. 2023; Rodrigues et al. 2022; Tuck and Yang 2012 for broader discussions on “decolonizing” fields). However, this conversation has become much more prominent, and now includes scientists from diverse backgrounds around the world (e.g., Adame 2023). Regardless of positionality, most participants agreed that if terms are exclusive, deterring entry to the field, or hurtful to anyone, then they should not be used. Given this position, the question remains: Who and how many individuals need to be bothered to make a case for the shift to newer terms?

I am Mexican, and I do not understand why I should label the natural riches of my country on the basis of the subjective perspective of colonizers five centuries ago. Fernanda Adame (2023)

## Why is language important to our conservation goals?

When we first began this discussion among smaller groups, the panelists wondered how much focus should be on language use when we are experiencing a primate extinction crisis. As multiple participants noted, counteracting the pernicious effects of a colonial legacy and conserving the world’s primates require increased opportunities for meaningful inclusion of local communities. Many of these communities still experience the legacy of colonialism and neocolonial practices in conservation-related discussions that exclude them (Dowie 2011; Lukumbo 2023; Pilisi 2023).

Language is a means of both inclusion and exclusion. In primatology, we have distinguished between habitat country/range country researchers and non-habitat/non-range country researchers. The term habitat/range country includes primatologists who are living/born in urban settings, with/without academic education, as well as Indigenous primatologists/conservationists, with/without academic education, and other nationals who are living/born locally near primate habitats (and may or may not have academic experience/training). These terms were likely coined by English-speaking scientists from the Global North. However, did anyone ask people from countries where wild populations of primates live if this is how they would want to be referred to? While range/habitat–country terminology came from well-intentioned goals of increasing funding and opportunities, these terms could also be considered exclusionary and may erase important nuances of individual backgrounds and opportunities (Adams et al. 2015). Researchers have not habitually distinguished who holds traditional knowledge, despite its importance for ecological and conservation goals. It is important not only to include traditional knowledge and the humans that hold this knowledge, but to engage fully with traditional knowledge, as we move toward a more inclusive conservation future (Gilio-Whitaker 2023).

## Conclusion

Participants in the IPS roundtable believe that all primatologists would agree that it is imperative to have Indigenous representation in our conservation initiatives. Yet, in primatology, Indigenous knowledge and concerns are rarely, if ever, explicitly included (but see Estrada et al. 2022; Shaffer et al. 2019; Urbani and Lizarralde 2020). In Nepal, when forests were “returned” to the Indigenous populations, they grew in size, biomass, and productivity (Kutter and Mitchell 2021). Many primatologists are recognizing that conservation has involved a tradition of social and environmental injustice (Brosius 2006; Dowie 2011; Pilisi 2023; Riley 2020; Tumusiime and Svarstad 2011 Water's et al. 2022), and many actions beyond changing language are needed to repair this damage.

The changing of words to make our fields more inclusive can be one step to increase participation among those we most want to welcome to our field and whose help is most needed to fight the extinction of primates and their habitats (Blair 2019; Garber et al. 2023). We need to listen to one another’s voices on this topic. Are we recommending changing the “Old World/New World” terminology? Although not unanimous, the majority of the panelists believe that this language should change, and it will happen organically as we update our teaching, grants, reports, publications, and outreach. While the majority of us agreed to change language, very few of us agreed that we need to prescribe new language. We do not have the authority to insist on new precise nomenclature and recognize that as scientists, we use terms that differ according to our research questions and area of study. Our conversation also revealed that expecting all primatologists to immediately change to an agreed-upon specific terminology (e.g., platyrrhines and catarrhines) would not be effective given different educational traditions, languages, and interpretation of the meanings of these words. We do not believe that there needs to be agreed-upon terms in order to replace offensive language. We can simply work to remove that language from our repertoires and do what is best to fit our agendas. We look forward to continuing conversations on this and related topics that inspire us all to reflect on the unintended consequences of our language use.
